# Editorial: New and old strategies to curb the obesity epidemic in children

**DOI:** 10.3389/fped.2024.1448039

**Published:** 2024-07-03

**Authors:** Valentina Fabiano, Valeria Calcaterra

**Affiliations:** ^1^ Department of Pediatrics, Vittore Buzzi Children’s Hospital, Milan, Italy; ^2^ Department of Biomedical and Clinical Sciences, University of Milan, Milan, Italy; ^3^ Department of Internal Medicine and Therapeutics, University of Pavia, Pavia, Italy

**Keywords:** children, adolescents, obesity, pediatric obesity, epidemics

**Editorial on the Research Topic** New and old strategies to curb the obesity epidemic in children

## Introduction

Obesity is defined as a condition characterized by an excess in body fat, which leads to an increased risk for morbidity and/or premature mortality (1). Childhood obesity is one of the most severe public health problems nowadays, and has become a global epidemic. The European Childhood Obesity Surveillance Initiative (COSI) reported a 29% prevalence of being overweight (including obese) in boys and 27% in girls aged 7–9 years; the prevalence of obesity was 13% in boys and 9% in girls (2).

The etiology of essential obesity is multifactorial, involving unhealthy eating patterns, a sedentary lifestyle, genetic and epigenetic influences, and environmental factors. These determinants of obesity act synergistically. It is well-known that obesity is associated with numerous multi-organ complications which, if not adequately recognized and treated, persist into adulthood, resulting in increased morbidity and mortality (3–5). Childhood obesity represents a serious public health challenge (2, 6) and curbing the obesity epidemics is a priority goal for the global health.

Healthy lifestyle strategies, such as physical activity and exercise, diet, and behavioral changes are crucial players to manage and prevent obesity and obesity-related conditions in children and adolescents (6), and a non-pharmacological treatment still remains the first line approach to obesity. However, lifestyle modification programs are not always successful.

In some selected cases, after the failure of a “conventional” approach, pharmacological or surgical treatment of obesity in pediatric patients can be applied (7–10).

However, despite all the efforts, to date, management of childhood obesity still remains a challenge. We launched this Frontiers Research Topic entitled “New and Old Strategies to Curb the Obesity Epidemics in Children” aiming to highlight the importance of continuing to study the determinants of disease and to explore the intricate relationship between obesity, lifestyle, and comorbidities, while simultaneously exploring new management options, including the use of technological innovation and digital health. Considering new and old strategies to curb obesity may be useful to offer the perspective of personalized preventive strategies and to propose innovative models for obesity treatment. The Research Topic also aimed supporting community intervention strategies and national/international interventions led by pivotal stakeholders.

## Summary of articles from this research topic

Five manuscripts were received for this Frontiers Research Topic. After rigorous review, four articles were finally accepted for publication. The contributing 23 authors were from five countries, including Italy, Mexico, United Kingdom, Portugal, and South Africa. This Research Topic received more than 6,400 views and 1,000 downloads as of June 2024. The key contents and findings of each paper are as follows:

### Ntimana et al.

In this systematic review, Ntimana et al. described the current literature on the determinants of central obesity and its associated health outcomes in children and adolescents in the South African population. The systematic review was conducted according to PRISMA guidelines. 11 studies were included in the review a total of 35,001 participants, of which 17,921 (51.2%) were boys and 17,080 (48.8%) were girls. The prevalence of central obesity in children and adolescents by waist-to-height ratio (WHtR) ranged from 2.0% to 41.0%; by waist-to-hip (WHR) ratio ranged from 10% to 25%; and by waist circumference (WC) ranged from 9% to 35%. According to several studies, central obesity in children and adolescents was determined by gender, age and pubertal development. Decreased physical activity and an increase in sedentarism behavior were also associated with central obesity in children, particularly in girls. Diet practices have also resulted as a contributing factor to central obesity. Last, children from families with high socioeconomic, and being in a household that is food secure were also associated with central obesity.

Data from this systematic review suggest the need for strategies, supported by healthcare providers, policymakers, and the general society aimed at reducing the burden of central obesity in South African children and adolescents.

### Porri et al.

In this review Porri et al. focused on the impact of different digital interventions for children and adolescent affected by obesity. The rapid technological and digital development, that has been incredibly pushed by the COVID-19 pandemic, has allowed an opening towards new therapeutic opportunities such as mobile healthcare. The digital and technological delivery of obesity prevention and treatment programs can represent an innovative tool to support children and families to access to obesity prevention programs. The implementation of telemedicine and text messages has demonstrated encouraging results in managing children and adolescents with obesity; similarly, the use of the mobile apps, both as a single strategy or in combination with traditional treatments, has shown a good efficacy level even if literature data derive from studies with different features. Web-based tools and social networks offer the opportunity for public health campaigns to raise awareness about obesity-related issues but also allow to support groups, online communities and individual to share their experiences, challenges and success in curbing against overweight and obesity. Moreover, the use of exergames, serious videogames and immersive gaming has also shown promising results in promoting healthy lifestyles for a successful weight management.

### Flores et al.

Monitoring child growth and development is important to enable the comparison of individual physical growth with reference population trends, and to analyze nutritional status. Differential height growth and velocity patterns of children and adolescents with normal-weight and overweight-obesity have been established in the United States and Europe; publications on child growth and modeling of the Mexican population are scarce. The study by Flores et al. aimed to estimate height growth velocity (of normal and overweight) of boys and girls by age, using the PB 1 model to understand whether overweight and normal-weight children and adolescents have different growth patterns and to develop normalized height growth curves for boys aged 2–18 years and girls aged 2–16 years using the LMS method and compare the results with the World Health Organization (WHO) growth standards and reference values to evaluate growth patterns by age and sex, if any. This study was the first of its kind that presented height growth velocity and centile curves of Mexican boys aged 2–18 years and girls aged 2–16 years. Mexican boys and girls experience timing and tempo of height growth at take-off and peak height velocity at an earlier age; the age at peak height velocity was earlier for overweight-obese children than normal weight-for-age peers in both sexes; and height values were different in the Mexican population as compared to the WHO's reference values at all ages. This last result may be related to a genetic or to a stunting condition that could not be identified in this study.

### Calcaterra et al.

In this review, Calcaterra et al. explored the intricate relationship between dietary habits, depression and obesity with the objective of elaborating preventive strategies for childhood obesity. Depression is a common mental health disorder whose multifaceted etiological mechanisms remain not completely understood. Obesity is characterized by hormonal imbalances and chronic inflammation that may play a role in precipitating depressive symptoms, and, on the other hand, depression may affect hormones involved in appetite-satiety circuit regulation. The relationship between the two is complex and bidirectional. Adolescence, typically characterized by psychosocial and physical changes, heightens the likelihood of co-occurring obesity and depression. This narrative review was conducted by making a comprehensive search of literature on the PubMed database and Cochrane Library, considering English language publications from the last 25 years. 89 full text papers were included in the review. Many studies have recognized a link between mood and food choices, with certain foods selected for their impact on the brain's reward centers. It is suggested that a specific neural mechanism exists for food addiction that contributes to overeating and obesity. The significant correlation between obesity and depression indicates a shared biological pathway. In addition, stress substantially affects eating behaviors, often leading to increased consumption of pleasurable and rewarding foods, specifically high in fat and calories food, triggering a cycle of overeating, weight gain, and psychological distress, exacerbating both mood disorders and obesity. However, studies in pediatric obesity and depression remain scarce, and there is actually insufficient evidence to drive clear conclusions about the possibility to carry out some therapeutically or preventive strategies in children and adolescence. Healthcare professionals, including mental health specialists, nutritionists and primary care providers, can provide support for a combination of psychotherapy, medication, lifestyle changes, and other healthcare issues. Integrated care, that addresses both mental and physical health, is crucial for effective management and long-term well-being.
